# Influenza vaccination and ischemic stroke risk reduction in elderly stroke survivors: a retrospective cohort study with negative control validation

**DOI:** 10.1186/s12877-025-06695-x

**Published:** 2025-11-28

**Authors:** Tianchi Yang, Hanwen Yang, Tianshu Zhou, Xiaoqing Wu, Lixia Ye

**Affiliations:** 1 Institute of Immunization and Prevention, Ningbo Municipal Center for Disease Control and Prevention, Ningbo, Zhejiang China; 2 School of Software, Henan University, Kaifeng, Henan China; 3 School of Public Health, Wenzhou Medical University, Wenzhou, Zhejiang China; 4 School of Public Health, Ningbo University, Ningbo, Zhejiang China

**Keywords:** Influenza vaccination, Ischemic stroke, Elderly stroke survivors, Retrospective cohort study, Competing risk models, Secondary prevention.

## Abstract

**Background and objectives:**

Stroke is a leading cause of disability and mortality among older adults, with high recurrence rates. Currently, there’s limited research reporting on the protective effect of influenza vaccination against recurrent stroke in elderly stroke survivors. This study aimed to evaluate the association between influenza vaccination and stroke risk in this group.

**Methods:**

A retrospective cohort study was conducted using data from the Ningbo Regional Health Information Platform. Eligible participants were adults aged ≥ 65 years with a history of stroke, registered in the platform for ≥ 1 year by December 31, 2021. Follow-up ended at stroke occurrence, death, or July 31, 2022, whichever occurred first. The primary outcome was any stroke event. Exposure was defined as influenza vaccination during the 2021–2022 season. Adjusted subdistribution hazard ratios (sHRs) and 95% confidence intervals (CIs) were estimated using Fine-Gray competing risk models. Pneumococcal vaccination was included as a negative control outcome.

**Results:**

Among 76,747 participants, 31,729 (41.3%) received influenza vaccination. Over 7 months, 6,323 stroke events occurred: 5,631 ischemic, 653 hemorrhagic, and 39 unspecified. After adjusting for demographics and comorbidities, influenza vaccination was associated with a reduced risk of ischemic stroke among elderly stroke survivors (particularly those whose last stroke event occurred ≥ 1 year prior) (adjusted sHR: 0.90, 95% CI: 0.85–0.95) but showed no significant association with hemorrhagic stroke risk (adjusted sHR: 0.89, 95% CI: 0.76–1.05). Additionally, influenza vaccination demonstrated significant protective effects against both cardiogenic stroke (adjusted sHR: 0.74, 95% CI: 0.63–0.86) and non-cardiogenic stroke (adjusted sHR: 0.93, 95% CI: 0.88–0.98), with a stronger protective effect observed for cardiogenic stroke.

**Conclusion:**

Influenza vaccination is associated with lower ischemic stroke risk among elderly stroke survivors, independent of cardiogenic etiology. These findings suggest a potential nonspecific preventive benefit and highlight the need for randomized trials to establish causality and inform clinical guidelines for stroke survivors.

**Supplementary Information:**

The online version contains supplementary material available at 10.1186/s12877-025-06695-x.

## Introduction

Stroke stands as a leading cause of mortality and long-term disability worldwide, with a particularly pronounced incidence and fatality rate among the elderly population [1, 2]. Stroke survivors face a substantially elevated risk of recurrence. Studies have demonstrated that more than 10% of individuals experience a recurrent stroke within one year following their initial event, and over one-quarter face a relapse within five years [3]. This exacerbates not only the economic burden on individuals and families but also poses a significant challenge to public health resources. As the global trend of aging intensifies, the effective management of stroke survivors’ health and the prevention of recurrent strokes have emerged as pressing issues demanding immediate attention [4].

Although numerous studies have investigated the association between influenza vaccination and stroke risk [5–9], research specifically focusing on elderly stroke survivors remains scarce. Furthermore, systematic reviews have revealed substantial heterogeneity across studies [5], with most existing research conducted in Western countries where vaccination status often relies on self-reported data [7]. Given regional disparities in stroke epidemiology, vaccination coverage, and healthcare systems, particularly in low- and middle-income countries in Asia and Africa—where stroke burden is high yet vaccination uptake is limited—there is an urgent need for rigorous, population-specific studies in these regions to generate additional evidence clarifying the potential protective benefits of influenza vaccination against stroke.

The present study aims to leverage the objective health-related data available through China’s sophisticated regional health information platforms to explore the impact of influenza vaccination on reducing the risk of stroke recurrence among elderly stroke survivors in China. We hypothesize that elderly stroke survivors who receive influenza vaccination will exhibit a significantly lower incidence of stroke recurrence compared to those who are unvaccinated. Considering that influenza vaccination coverage among the elderly in many countries globally still falls far below the World Health Organization’s recommended 75% [10], if our findings support this hypothesis, promoting influenza vaccination could emerge as an effective and economical strategy for preventing stroke recurrence in elderly stroke survivors. This could profoundly impact the improvement of elderly individuals’ quality of life and the alleviation of burdens on healthcare systems.

## Method

### Study design and setting

This study is a retrospective observational cohort study designed to investigate the impact of influenza vaccination on the risk of stroke recurrence among elderly stroke survivors. The data utilized in this study were sourced from the Ningbo Regional Health Information Platform (RHIP), which encompasses medical records, vaccination records, and death registration information. Ningbo is a coastal metropolis in southeast China with a developed economy and a population exceeding 9 million. It boasts a comprehensive population health information system that achieved the highest level of interconnection certification from the National Health Commission of China in 2016. The unique identity numbers assigned to residents facilitate seamless medical access, vaccination tracking, and death registration, enabling the platform to maintain detailed individual records using advanced record linkage techniques. The reliability and accuracy of this data source have been thoroughly validated through multiple studies [11–13].

The study was approved by the Ethics Committee of Ningbo Municipal Center for Disease Control and Prevention, China (IRB. No: 202208). Due to the anonymous and population-based nature of the study, the Ethics Committee of Ningbo Municipal Center for Disease Control and Prevention granted a waiver of informed consent.

### Study population

The study population was defined as individuals aged 65 and older, with local residency, and a history of stroke, who were still alive as of December 31, 2021. Inclusion criteria further required that these individuals had been registered on the Regional Health Information Platform for at least one year prior to the study initiation and had at least one medical visit record between January and July 2022. Participants meeting these criteria were considered eligible for inclusion in the study. Additionally, patients diagnosed with stroke between October 1, 2021, and December 31, 2021, or those who received influenza vaccination between January 1, 2022, and July 31, 2022, were excluded from the study.

### Exposure variables, outcome variables, and other covariates

In this study, the primary exposure factor of interest is the influenza vaccination received during the 2021–2022 influenza season. Beginning with the 2021–2022 season, the government in the study region initiated a funded program aimed at providing free trivalent inactivated influenza vaccines to elderly residents aged 65 and over with local household registration. Given that attenuated influenza vaccines have not yet gained approval for use in adults in China, our study specifically focuses on inactivated vaccines, encompassing both trivalent and quadrivalent vaccines. For the purposes of this study, the exposed group is defined as individuals who underwent seasonal influenza vaccination between August 1, 2021, and December 31, 2021, with the vaccination date occurring at least 14 days before the event of interest or the termination of the observation [14, 15]. Other individuals are considered as the non-exposed group.

The outcome of interest in this study is any stroke event, including stroke-related deaths. Stroke diagnoses were based on primary clinical diagnosis codes from the International Classification of Diseases, Tenth Revision (ICD-10), and were further categorized into hemorrhagic (codes I60.x, I61.x, and I62.x), ischemic (code I63.x), and unclassified (code I64.x) strokes. Additionally, strokes were stratified by etiology into cardiogenic (codes I63.1, I63.4) and non-cardiogenic (codes I60, I61, I62, and other I63.x codes except for I63.1 and I63.4) subtypes. For each participant, the observation period began on January 1, 2022, and ended upon stroke occurrence, death, or July 31, 2022, whichever came first.

Potential relevant covariates encompassed age, sex, current residential address categorized as urban or suburban based on administrative division codes, history of pneumococcal vaccination within the past five years (specifically, the 23-valent pneumococcal polysaccharide vaccine, which is currently the only pneumococcal vaccine available for adults in China), and underlying disease status such as hypertension, diabetes, coronary artery disease (CAD), malignant tumors, chronic obstructive pulmonary disease (COPD), and atrial fibrillation.

All of the above data were obtained from the data warehouse of the RHIP in the study area.

### Statistical analyses

In our study, we conducted statistical descriptions of the distribution and characteristics of study participants based on their influenza vaccination status. For categorical variables, descriptive statistics were presented using counts and percentages, with Chi-square tests employed for comparisons between groups. For continuous variables, descriptive statistics were provided as means ± standard deviations or medians (with interquartile ranges), and group comparisons were conducted using t-tests or Wilcoxon rank-sum tests, depending on the distribution of the data.

To assess the independent effect of influenza vaccination on the risk of stroke incidence, we utilized the Fine-Gray competing risks model, with results expressed as subdistribution hazard ratios (HRs) and their corresponding 95% confidence intervals (CIs). In analyzing the risk of stroke onset, individuals who did not experience a stroke were considered as censored observations, whereas stroke onset (including stroke-related deaths) and deaths from other causes were defined as competing events. The proportional hazards assumption was assessed in our study (see Supplementary Table 1). The final multivariate model included variables with p-values less than 0.10 in univariate analysis that also met the proportional hazards assumption. For covariates that did not satisfy the proportional hazards assumption, stratified analyses were conducted, including: (1) type of residence, (2) history of CAD, (3) subtype of prior stroke, and (4) time interval since last stroke. Furthermore, sensitivity analyses were conducted, which included: (1) employing a propensity score matching methods; (2) excluding cases diagnosed with influenza or influenza-like illness (ILI) between June 1, 2021, and December 31, 2021 (the definition of ILI is provided in Supplementary Table 2); (3) excluding cases of atrial fibrillation; and (4) using pneumococcal vaccination as a negative control.

All statistical analyses were conducted using R software, version 4.2.2. A two-sided P-value less than 0.05 was considered statistically significant.

## Result

### Enrollment

**Fig. 1:**
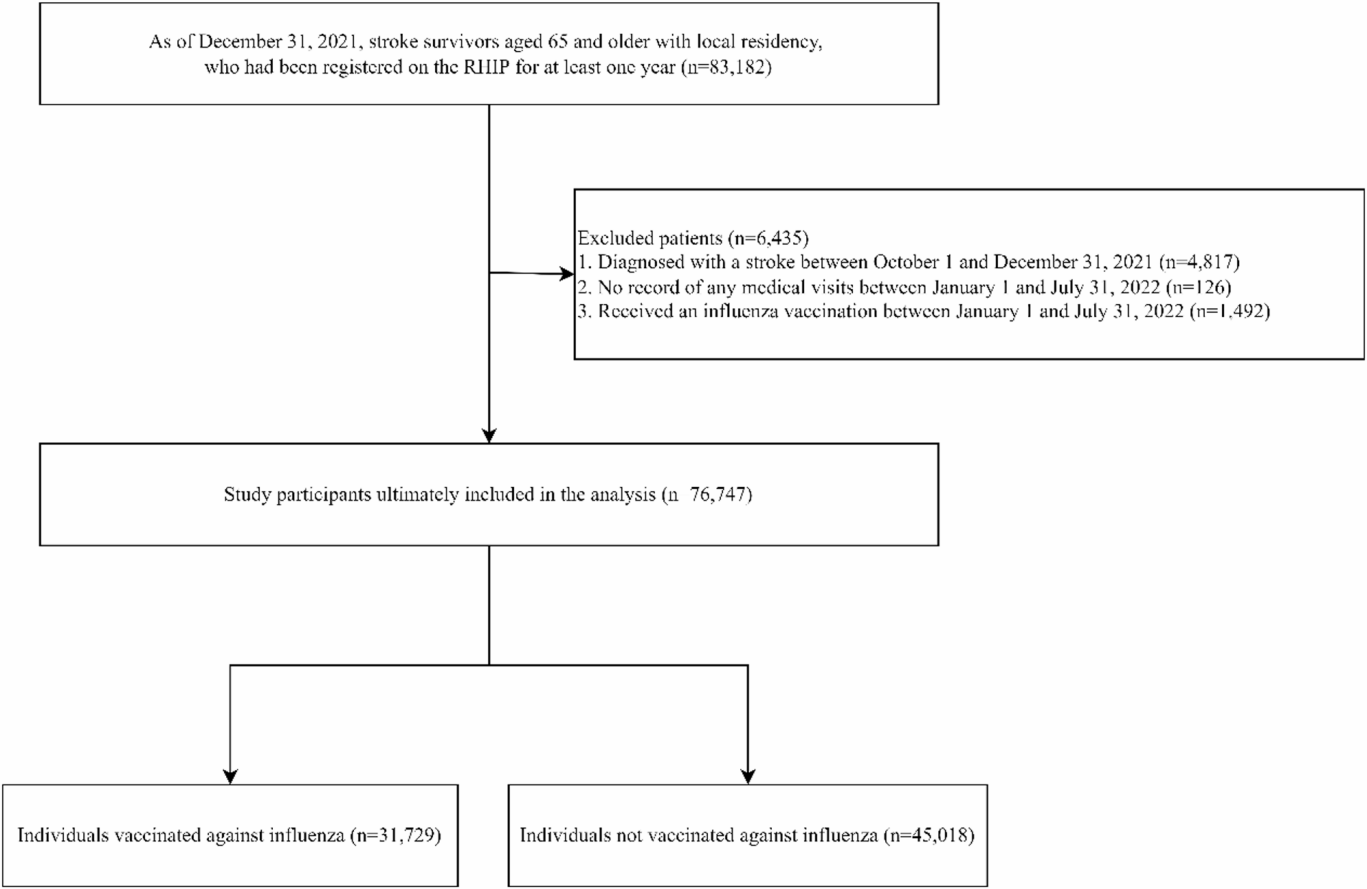
Flow Diagram for Study Enrollment

By December 31, 2021, we identified 83,182 stroke survivors aged ≥ 65 with local residency and ≥ 1 year of RHIP registration. Applying strict inclusion and exclusion criteria, 76,747 were included in the final analysis (Fig. 1).

### Baseline characteristics

A total of 31,729 participants received the seasonal influenza vaccination, among which 98.2% were administered the trivalent inactivated vaccine, and 1.8% received the quadrivalent inactivated vaccine. Over the subsequent seven months, 6,323 cases of visits due to stroke were observed. Of these strokes, 89.1% (5,631/6,323) were ischemic, 10.3% (653/6,323) were hemorrhagic, and 0.6% (39/6,323) were unclassified. Additionally, 799 deaths attributable to causes other than stroke were recorded. Baseline characteristics of the patients are presented in Table 1.

**Table 1:** Baseline demographic and clinical characteristics of study participants

Characteristics	*n* (%)
Total	76,747 (100)
Age, years, M (IQR)	76 (11)
Age group	
65–70 years	15,524 (20.2)
70–75 years	18,273 (23.8)
75–80 years	17,336 (22.6)
≥ 80 years	25,614 (33.4)
Sex	
Female	37,177 (48.4)
Male	39,570 (51.6)
Type of residence	
Urban	30,730 (40.0)
Suburban	46,017 (60.0)
Type of stroke history	
Hemorrhagic	10,604 (13.8)
Ischemic	66,023 (86.0)
Unclassified	120 (0.2)
Time since last stroke	
<1 year	17,798 (23.2)
1–2 years	21,033 (27.4)
≥2 years	37,916 (49.4)
Underlying disease status	
Hypertension	35,912 (46.8)
Diabetes	11,639 (15.2)
CAD	5,963 (7.8)
Tumor	1,066 (1.4)
COPD	3,121 (4.1)
Atrial fibrillation	8,745 (11.4)
Pneumococcal vaccination	
Unvaccinated	75,413 (98.3)
Vaccinated	1,334 (1.7)
Influenza vaccination status	
Unvaccinated	45,018 (58.7)
Trivalent vaccine	31,152 (40.6)
Quadrivalent vaccine	577 (0.7)
Survival outcome	
Stroke	6,323 (8.2)
Non-stroke deaths	799 (1.0)
Censoring	69,625 (90.7)
Stroke subtypes	
Hemorrhagic	653 (0.9)
Ischemic	5,631 (7.3)
Unclassified	39 (0.1)
Etiological subtypes	
Cardiac	858 (1.1)
Non-cardiac	5,426 (7.1)

*Abbreviations* *M (IQR)* median (interquartile range), *CAD* coronary artery disease, *COPD* chronic obstructive pulmonary disease

There were significant statistical differences between influenza vaccine recipients and non-recipients in terms of age, type of residence, type of last stroke and its interval, receipt of a pneumococcal vaccine within the past five years, and prevalence of hypertension, diabetes, tumor, COPD, and atrial fibrillation. Specifically, recipients were more likely to be 70 years or older, reside in urban areas, have a history of hemorrhagic stroke, have experienced a stroke more than a year ago, have received a pneumococcal vaccine within the past five years, and have hypertension, diabetes, tumor, COPD, or no history of atrial fibrillation. These detailed findings are summarized in Table 2. In univariate analysis (see Supplementary Table 3), being under tumor management and having received a pneumococcal vaccine within the past five years were not significantly associated with the risk of recurrent stroke (*P* > 0.05), while other baseline characteristics were significantly correlated with stroke recurrence (*P* < 0.05).

**Table 2:** Comparison of baseline characteristics between participants with and without influenza vaccination

Characteristics	Influenza vaccination status, *n* (%)		*P* value^*^
	Vaccinated	Unvaccinated	
Total	31,729 (41.3)	45,018 (58.7)	
Age, years, M (IQR)	75 (9)	76 (14)	< 0.001
Age group			< 0.001
65–70 years	5,122 (16.1)	10,402 (23.1)	
70–75 years	9,465 (29.8)	8,808 (19.6)	
75–80 years	9,007 (28.4)	8,329 (18.5)	
≥ 80 years	8,135 (25.6)	17,479 (38.8)	
Sex			0.525
Female	15,326 (48.3)	21,851 (48.5)	
Male	16,403 (51.7)	23,167 (51.5)	
Type of residence			< 0.001
Urban	13,653 (43.0)	17,077 (37.9)	
Suburban	18,076 (57.0)	27,941 (62.1)	
Type of stroke history			< 0.001
Hemorrhagic	4,720 (14.9)	5,884 (13.1)	
Ischemic	26,987 (85.1)	39,036 (86.7)	
Unclassified	22 (0.1)	98 (0.2)	
Time since last stroke			< 0.001
<1 year	6,954 (21.9)	10,844 (24.1)	
1–2 years	9,428 (29.7)	11,605 (25.8)	
≥2 years	15,347 (48.4)	22,569 (50.1)	
Underlying disease status			
Hypertension	17,169 (54.1)	18,743 (41.6)	< 0.001
Diabetes	5,416 (17.1)	6,223 (13.8)	< 0.001
CAD	2,486 (7.8)	3,477 (7.7)	0.579
Tumor	513 (1.6)	553 (1.2)	< 0.001
COPD	1,416 (4.5)	1,705 (3.8)	< 0.001
Atrial fibrillation	3,262 (10.3)	5,483 (12.2)	< 0.001
Pneumococcal vaccination			< 0.001
Unvaccinated	30,687 (96.7)	44,726 (99.4)	
Vaccinated	1,042 (3.3)	292 (0.6)	

*Abbreviations* *M (IQR)* median (interquartile range), *CAD* coronary artery disease, *COPD* chronic obstructive pulmonary disease

^*^Categorical variables were analyzed using either the Pearson χ² test or Fisher’s exact test, depending on the sample size and expected frequencies. For continuous variables, the Wilcoxon rank sum test (for non-parametric data or when the assumptions of the t-test were violated) or the t-test (for parametric data meeting the necessary assumptions) was employed

## Association between influenza vaccination and stroke incidence

The univariate analysis revealed a statistically significant association between influenza vaccination and stroke incidence, with an sHR of 0.84 (95% CI: 0.80–0.89). This correlation remained statistically significant in the multivariate analysis after adjusting for potential confounding factors such as age, sex, status of chronic underlying conditions, and history of pneumococcal vaccination, with an adjusted sHR of 0.90 (95% CI: 0.85–0.95). Further analysis of stroke subtypes indicated that influenza vaccination was associated with a decreased incidence of ischemic stroke, with an adjusted sHR of 0.90 (95% CI: 0.85–0.95). In contrast, no statistically significant impact was observed on the risk of hemorrhagic stroke, with an adjusted sHR of 0.89 (95% CI: 0.76–1.05). When stratified by stroke etiology, influenza vaccination demonstrated significant protective effects against both cardiogenic stroke (adjusted sHR: 0.74, 95% CI: 0.63–0.86) and non-cardiogenic stroke (adjusted sHR: 0.93, 95% CI: 0.88–0.98), with a stronger protective effect observed for cardiogenic stroke. These results are summarized in Table 3.

**Table 3:** Association between influenza vaccination and stroke incidence during 7-Month Follow-Up

Outcomes	Influenza vaccination status, *n* (%)		sHR (95% CI)	
	Vaccinated	Unvaccinated	Crude	Adjusted^*^
Stroke recurrence	2,365 (7.45)	3,958 (8.79)	0.84 (0.80–0.89)	0.90 (0.85–0.95)
Stroke subtypes				
Hemorrhagic	227 (0.77)	426 (1.03)	0.75 (0.64–0.88)	0.89 (0.76–1.05)
Ischemic	2,131 (6.77)	3,500 (7.85)	0.86 (0.81–0.90)	0.90 (0.85–0.95)
Etiological subtypes				
Cardiac	247 (0.83)	611 (1.47)	0.57 (0.49–0.66)	0.74 (0.63–0.86)
Non-cardiac	2,111 (6.71)	3,315 (7.47)	0.89 (0.85–0.94)	0.93 (0.88–0.98)

*Abbreviations* *sHR* subdistribution hazard ratio, *CI* confidence interval

^*^Adjusted for various covariates including age group, sex, hypertension, diabetes, tumor, COPD, atrial fibrillation, and pneumococcal vaccination status

The results of stratified analyses demonstrated that there were no statistically significant interactions between influenza vaccination and the incidence of ischemic stroke with respect to type of residence, history of CAD, or subtype of previous stroke (all P values for interaction > 0.05). However, a significant interaction was observed for the time interval since the last stroke episode. Specifically, influenza vaccination provided protective effects against subsequent ischemic stroke, particularly in patients who experienced a stroke more than one year prior. These findings are presented in Table 4.

**Table 4:** Association between influenza vaccination and risk of ischemic stroke: stratified analysis by residence Type, history of coronary artery Disease, prior stroke Subtype, and time interval since last stroke

Characteristics	Influenza vaccination, *n* (%)		Adjusted sHR (95% CI) ^*^	*P* for interaction
	Vaccinated	Unvaccinated		
Type of residence				0.42
Urban	624 (4.60)	916 (5.41)	0.90 (0.81-1.00)	
Suburban	1,507 (8.41)	2,584 (9.36)	0.94 (0.88-1.00)	
History of CAD				0.91
No	1,976 (6.81)	3,253 (7.91)	0.90 (0.85–0.96)	
Yes	155 (6.26)	247 (7.15)	0.91 (0.75–1.12)	
Subtype of prior stroke				
Hemorrhagic	59 (1.30)	100 (1.81)	0.71 (0.51-1.00)	Ref
Ischemic	2,071 (7.69)	3,399 (8.73)	0.92 (0.87–0.98)	0.22
Unclassified	1 (6.67)	1 (1.37)	3.04 (0.02–504)	0.17
Time interval since last stroke				
< 1 year	895 (12.90)	1,402 (13.10)	0.98 (0.90–1.07)	Ref
1–2 years	461 (4.90)	672 (5.82)	0.88 (0.77–0.99)	0.046
≥ 2 years	775 (5.11)	1,426 (6.40)	0.90 (0.82–0.99)	< 0.001

*Abbreviations* *CAD* coronary artery disease, *sHR* subdistribution hazard ratio, *CI* confidence interval

^*^Adjusted for the covariates including age group, sex, hypertension, diabetes, tumor, COPD, atrial fibrillation, and pneumococcal vaccination status

### Sensitivity analysis

In sensitivity analyses (Supplementary Tables 4, 5 and 6), the findings remained consistent with the primary analysis results regardless of whether propensity score matching method was applied, cases diagnosed with influenza or ILI between June 1st and December 31st, 2021 were excluded, or individuals with a history of atrial fibrillation were excluded. Furthermore, in contrast to influenza vaccination, pneumococcal vaccination did not exhibit a comparable protective effect against stroke (adjusted sHR: 0.96, 95% CI: 0.78–1.17) (Supplementary Table 7).

## Discussion

In this large-scale retrospective cohort study, we demonstrated a significant association between influenza vaccination and a reduced risk of ischemic stroke in elderly stroke survivors, particularly among those whose most recent stroke occurred ≥ 1 year prior. Further analysis revealed that the protective effect of influenza vaccination against stroke was not restricted by whether the stroke was of cardiogenic etiology, with a more pronounced effect observed for cardiogenic stroke. These findings align with prior reports [5, 7, 16]but extend them by elucidating the specific scope of influenza vaccine’s protective effects in secondary stroke prevention. Notably, the 10% risk reduction observed—while modest in magnitude—carries substantial population-level implications given that ischemic stroke constitutes 80–90% of all stroke events [17], the devastating consequences of recurrence [3], and the persistent underutilization of influenza vaccination in most countries [10]. Together, these factors underscore the vaccine’s untapped potential as a cost-effective adjunct to conventional stroke prevention strategies, warranting reevaluation of clinical guidelines and public health immunization policies.

A recently published meta-analysis reported robust reductions in stroke incidence across subtypes—ischemic (OR = 0.77, 95% CI: 0.72–0.82), hemorrhagic (OR = 0.80, 95% CI: 0.72–0.89), and unspecified (OR = 0.83, 95% CI: 0.77–0.89)—among vaccinated adults [5]. However, the authors acknowledged significant heterogeneity across included studies. Our analysis did not observe a protective effect against hemorrhagic stroke, consistent with several null findings reported in the literature [9, 18–20]. Methodological discrepancies likely underlie these contradictions, including differences in cohort selection (primary vs. secondary prevention populations), analytical approaches, and adjustment for time-varying confounders. Collectively, these observations underscore the critical need for additional high-quality studies to resolve these inconsistencies.

The precise mechanisms underlying the potential benefits of influenza vaccination in reducing the risk of ischemic stroke remain an enigma. Despite this, several credible and plausible explanations have been proposed and investigated. A primary hypothesis centers on the prevention of influenza infection itself. Influenza can elicit immune responses that result in a procoagulant state, promoting atherosclerosis and subsequently increasing the risk of ischemic stroke [21, 22]. By preventing influenza infection at its inception, influenza vaccination could potentially disrupt this chain of events. However, Rodriguez - Martin et al. reported that influenza vaccination exhibited a protective effect against stroke even prior to the influenza pandemic [23], a phenomenon that cannot be solely explained by the vaccine’s protection against influenza infection. An alternative explanation suggests that the protective effect of influenza vaccination against ischemic stroke may not only be attributed to the prevention of influenza virus infection itself but may also be related to broader, nonspecific immunomodulatory effects, such as the reduction of proinflammatory cytokines and the exertion of anti-inflammatory, anticoagulant, and plaque-stabilizing properties [8, 23]. Our study found that although the protective effect of influenza vaccination was more pronounced for cardiogenic stroke, its protective effect was not restricted by cardiogenic etiology, meaning it was not solely targeted at thrombus formation related to atrial fibrillation. This supports the notion that the vaccine’s mechanism of action is universal. Therefore, we lean towards the latter hypothesis, namely, that the protective effect of influenza vaccination against ischemic stroke is more likely mediated through nonspecific pathways. Certainly, some researchers argue that the protective effect of influenza vaccination against stroke could be due to unmeasured confounding factors [8, 23]. However, if this were the case, one would expect similar stroke prevention effects from pneumococcal polysaccharide vaccination due to unmeasured confounding. Yet, neither our study nor previously reported research has shown similar stroke prevention effects for pneumococcal polysaccharide vaccination within the same research framework [23, 24]. Future studies are needed to further elucidate the mechanisms underlying this association and to confirm our findings in larger, more diverse populations.

Benefiting from the well-established regional health information platform in our study area, the medical records, vaccination histories, and mortality data we obtained were sourced from objective records of real-world medical activities. This not only ensured the comprehensiveness of our data but also imparted a high degree of reliability, thereby minimizing recall bias and significantly reducing the potential for misclassification of vaccination status and case outcomes. These reliable data formed the cornerstone of our analysis. Furthermore, another advantage of our study lies in its broad sample coverage, encompassing all elderly stroke survivors within the entire region, thereby enhancing the representativeness and generalizability of our findings. We also addressed the competing risk of mortality and adjusted for potential confounding factors that could influence the study results, striving to ensure the scientific rigor and precision of our analysis. Additionally, we conducted subgroup analyses based on stroke subtypes and etiologies to explore the potential mechanisms underlying the vaccine’s impact on stroke. However, our study is not devoid of notable limitations. Firstly, given that the data were derived from administrative datasets, we were unable to directly obtain information on participants’ personal habits (such as smoking and alcohol consumption habits, as well as frailty levels). Additionally, data protection policies restricted our access to information on medication use, including anticoagulant therapy. The absence of these data may have impacted our interpretation of the results to some extent. To mitigate this limitation, we employed chronic underlying disease status as a surrogate indicator of health and conducted a supplementary sensitivity analysis among individuals without a history of atrial fibrillation to verify the robustness of the conclusions. Secondly, specific populations were excluded from the analysis, including stroke patients who sought medical attention outside the study area and those who experienced a stroke but did not seek medical care. This could introduce selection bias. However, it is worth noting that due to the urgency and severity of stroke, coupled with the efficient and accessible healthcare system in the study area, residents typically seek timely medical attention nearby after onset, which greatly alleviates the potential impact of selection bias. Lastly, our study employed a retrospective cohort design, inherently limiting our ability to establish causality. Therefore, it is imperative to conduct further randomized controlled trials to validate our conclusions.

In conclusion, the study revealed that influenza vaccination can decrease the risk of ischemic stroke onset among elderly stroke survivors, and this protective effect is not restricted by cardiogenic etiologies. These findings suggest that influenza vaccination holds promise as an effective means for preventing ischemic stroke risk in this patient population. It is recommended to conduct randomized controlled trials to confirm these observational results.

## Supplementary Information

Supplementary Material 1.

## Data Availability

Please contact the corresponding author for data requests.

## Abbreviations

RHIP: Regional health information platform
ICD-10: International classification of diseases, tenth revision
CAD: Coronary artery disease
COPD: Chronic obstructive pulmonary disease
HR: Hazard ratio
CI: Confidence interval
ILI: Influenza-like illness
