# Supplementary material for: Influenza vaccination and ischemic stroke risk reduction in elderly stroke survivors: a retrospective cohort study with negative control validation
Source: BMC Geriatr. 2025 Nov 28;25:984. doi: 10.1186/s12877-025-06695-x (PMC12664219; doi:10.1186/s12877-025-06695-x)
Supplement: Supplementary file 1 — Supplementary Material 1. [file 12877_2025_6695_MOESM1_ESM.docx]

Supplementary Table 1. Assessment of Proportional Hazards Assumption Using Global Test Statistic

Supplementary Table 2. ICD-10 Codes for Definition of "Influenza-Like Illness"

Supplementary Table 3. Univariate Analysis of Baseline Characteristics and Stroke Risk Using Fine-Gray Competing Risks Model

Supplementary Table 4. Association Between Influenza Vaccination and Stroke Incidence During 7-Month Follow-Up: Propensity Score-Matched Analysis

Supplementary Table 5. Association Between Influenza Vaccination and Stroke Incidence During 7-Month Follow-Up After Excluding Participants Diagnosed with Influenza or ILI Between June 1, 2021, and December 31, 2021

Supplementary Table 6. Association Between Influenza Vaccination and Stroke Incidence During 7-Month Follow-Up After Excluding Individuals With a Prior History of Atrial Fibrillation

Supplementary Table 7. Association Between Pneumococcal Vaccination and Stroke Incidence During 7-Month Follow-Up

Supplementary Table 1. Assessment of Proportional Hazards Assumption Using Global Test Statistic

| Variables | Scenario 1 | | | |  | Scenario 2 | | | |
| --- | --- | --- | --- | --- | --- | --- | --- | --- | --- |
|  | Inclusion | chisq | df | p |  | Inclusion | chisq | df | p |
| Age group | ✓ | 7.5951 | 3 | 0.055 |  | ✓ | 7.3988 | 3 | 0.060 |
| Sex | ✓ | 1.7881 | 1 | 0.181 |  | ✓ | 1.8103 | 1 | 0.178 |
| Type of residence | ✓ | 37.3985 | 1 | 9.6e-10 |  | 🗶 |  |  |  |
| Type of stroke history | ✓ | 22.2656 | 2 | 1.5e-05 |  | 🗶 |  |  |  |
| Time since last stroke | ✓ | 91.8691 | 2 | <2e-16 |  | 🗶 |  |  |  |
| Hypertension | ✓ | 2.6225 | 1 | 0.105 |  | ✓ | 2.7777 | 1 | 0.096 |
| Diabetes | ✓ | 0.4947 | 1 | 0.482 |  | ✓ | 0.5383 | 1 | 0.463 |
| CAD | ✓ | 4.3731 | 1 | 0.037 |  | 🗶 |  |  |  |
| Tumors | ✓ | 1.6025 | 1 | 0.206 |  | ✓ | 1.5833 | 1 | 0.208 |
| COPD | ✓ | 0.5942 | 1 | 0.441 |  | ✓ | 0.6072 | 1 | 0.436 |
| Atrial fibrillation | ✓ | 0.0061 | 1 | 0.938 |  | ✓ | 0.0121 | 1 | 0.912 |
| Pneumococcal vaccination | ✓ | 0.0832 | 1 | 0.773 |  | ✓ | 0.0659 | 1 | 0.797 |
| Influenza vaccination | ✓ | 0.7061 | 1 | 0.401 |  | ✓ | 0.7052 | 1 | 0.401 |
| GLOBAL |  | 148.3724 | 17 | <2e-16 |  |  | 16.5764 | 11 | 0.121 |

Abbreviations: CAD, coronary artery disease; COPD, chronic obstructive pulmonary disease.

Supplementary Table 2. ICD-10 Codes for Definition of "Influenza-Like Illness"

| Diseases | ICD codes |
| --- | --- |
| Cold | J00 |
| Sinusitis | J01, J32 |
| Pharyngitis | J02 |
| Laryngitis, tracheitis or laryngotracheitis | J04 |
| Upper respiratory tract infections | J06.8 or J06.9 |
| Influenza | J09-J11 |
| Pneumonia | J12-J18 |
| Acute bronchitis, bronchiolitis (not specifically acute or chronic), obstructive bronchitis | J20, J40, J44.8 |
| Fine bronchitis, capillary bronchitis | J21 |
| Acute lower respiratory tract infections, nonspecific | J22 |
| Chronic obstructive pulmonary disease combined with acute lower respiratory tract infection | J44.0 |
| Chronic obstructive pulmonary disease with acute exacerbation | J44.1 |
| Cough | R05 |
| Pleurisy | R09.1 |

Abbreviations: ICD, International Classification of Diseases.

Supplementary Table 3. Univariate Analysis of Baseline Characteristics and Stroke Risk Using Fine-Gray Competing Risks Model

| Characteristics | sHR (95% CI) | *P* value |
| --- | --- | --- |
| Age group |  |  |
| 65-70 years | Ref |  |
| 70-75 years | 1.26 (1.15-1.37) | <0.001 |
| 75-80 years | 1.49 (1.37-1.62) | <0.001 |
| ≥80 years | 1.92 (1.78-2.07) | <0.001 |
| Sex |  |  |
| Female | Ref |  |
| Male | 1.16 (1.11-1.22) | <0.001 |
| Type of residence |  |  |
| Suburban | Ref |  |
| Urban | 0.56 (0.53-0.59) | <0.001 |
| Type of stroke history |  |  |
| Hemorrhagic | Ref |  |
| Ischemic | 1.35 (1.25-1.46) | <0.001 |
| Unclassified | 4.71 (3.41-6.49) | <0.001 |
| Time since last stroke |  |  |
| <1 year | Ref |  |
| 1-2 years | 0.40 (0.37-0.43) | <0.001 |
| ≥2 years | 0.49 (0.46-0.52) | <0.001 |
| Underlying disease status |  |  |
| Hypertension | 0.67 (0.64-0.71) | <0.001 |
| Diabetes | 0.80 (0.75-0.86) | <0.001 |
| CAD | 0.87 (0.79-0.96) | <0.001 |
| Tumors | 0.95 (0.77-1.18) | 0.65 |
| COPD | 1.23 (1.10-1.38) | <0.001 |
| Atrial fibrillation‌ | 1.23 (1.15-1.32) | <0.001 |
| Pneumococcal vaccination | 0.91 (0.75-1.11) | 0.38 |
| Influenza vaccination | 0.84 (0.80-0.89) | <0.001 |

Abbreviations: CAD, coronary artery disease; COPD, chronic obstructive pulmonary disease; sHR, subdistribution hazard ratio; CI, confidence interval.

Supplementary Table 4. Association Between Influenza Vaccination and Stroke Incidence During 7-Month Follow-Up: Propensity Score-Matched Analysis (n=61400)

| Outcomes | Influenza vaccination status, n (%) | | sHR (95% CI) |
| --- | --- | --- | --- |
|  | Vaccinated | Unvaccinated |  |
| Stroke recurrence | 2305 (7.51) | 2458 (8.01) | 0.94 (0.88-0.99) |
| Stroke subtypes |  |  |  |
| Hemorrhagic | 225 (0.79) | 228 (0.80) | 0.98 (0.82-1.18) |
| Ischemic | 2074 (6.81) | 2221 (7.29) | 0.93 (0.88-0.99) |
| Etiological subtypes |  |  |  |
| Cardiac | 243 (0.85) | 312 (1.09) | 0.78 (0.66-0.92) |
| Non-cardiac | 2056 (6.75) | 2137 (7.03) | 0.96 (0.90-1.02) |

Abbreviations: sHR, subdistribution hazard ratio; CI, confidence interval.

Supplementary Table 5. Association Between Influenza Vaccination and Stroke Incidence During 7-Month Follow-Up After Excluding Participants Diagnosed with Influenza or ILI Between June 1, 2021, and December 31, 2021 (n=67418)

| Outcomes | Influenza vaccination status, n (%) | |  | sHR (95% CI) | |
| --- | --- | --- | --- | --- | --- |
|  | Vaccinated | Unvaccinated |  | Crude | Adjusted^*^ |
| Stroke recurrence | 2015 (7.33) | 3501 (8.77) |  | 0.83 (0.79-0.88) | 0.89 (0.84-0.94) |
| Stroke subtypes |  |  |  |  |  |
| Hemorrhagic | 195 (0.76) | 391 (1.06) |  | 0.71 (0.60-0.85) | 0.86 (0.72-1.02) |
| Ischemic | 1814 (6.65) | 3082 (7.80) |  | 0.85 (0.80-0.90) | 0.89 (0.84-0.95) |
| Etiological subtypes |  |  |  |  |  |
| Cardiac | 218 (0.85) | 548 (1.48) |  | 0.57 (0.49-0.67) | 0.75 (0.64-0.88) |
| Non-cardiac | 1791 (6.57) | 2925 (7.43) |  | 0.88 (0.83-0.93) | 0.91 (0.86-0.97) |

Abbreviations: sHR, subdistribution hazard ratio; CI, confidence interval.

^*^Adjusted for various covariates including age group, sex, underlying disease status (hypertension, diabetes, tumor, COPD, and atrial fibrillation), and pneumococcal vaccination status.

Supplementary Table 6. Association Between Influenza Vaccination and Stroke Incidence During 7-Month Follow-Up After Excluding Individuals With a Prior History of Atrial Fibrillation (n=68002)

| Outcomes | Influenza vaccination status, n (%) | |  | sHR (95% CI) | |
| --- | --- | --- | --- | --- | --- |
|  | Vaccinated | Unvaccinated |  | Crude | Adjusted^*^ |
| Stroke recurrence | 2059 (7.23) | 3407 (8.62) |  | 0.83 (0.79-0.88) | 0.89 (0.84-0.94) |
| Stroke subtypes |  |  |  |  |  |
| Hemorrhagic | 199 (0.75) | 381 (1.04) |  | 0.72 (0.60-0.85) | 0.86 (0.72-1.03) |
| Ischemic | 1854 (6.56) | 2997 (7.66) |  | 0.85 (0.80-0.90) | 0.90 (0.84-0.95) |
| Etiological subtypes |  |  |  |  |  |
| Cardiac | 211 (0.79) | 520 (1.42) |  | 0.56 (0.47-0.65) | 0.72 (0.61-0.85) |
| Non-cardiac | 1842 (6.52) | 2858 (7.33) |  | 0.89 (0.84-0.94) | 0.92 (0.86-0.97) |

Abbreviations: sHR, subdistribution hazard ratio; CI, confidence interval.

^*^Adjusted for various covariates including age group, sex, underlying disease status (hypertension, diabetes, tumor, COPD), and pneumococcal vaccination status.

Supplementary Table 7. Association Between Pneumococcal Vaccination and Stroke Incidence During 7-Month Follow-Up

| Outcome | Pneumococcal vaccination status, n (%) | |  | sHR (95% CI) | |
| --- | --- | --- | --- | --- | --- |
|  | Vaccinated | Unvaccinated |  | Crude | Adjusted^*^ |
| Stroke recurrence | 101 (7.57) | 6222 (8.25) |  | 0.91 (0.75-1.11) | 0.96 (0.78-1.17) |

Abbreviations: sHR, subdistribution hazard ratio; CI, confidence interval.

^*^Adjusted for various covariates including age group, sex, underlying disease status (hypertension, diabetes, tumor, COPD, and atrial fibrillation), and pneumococcal vaccination status.
